# Characterization of household microbiomes from three unique cities around the world

**DOI:** 10.1099/mgen.0.001861

**Published:** 2026-11-06

**Authors:** Caroline Scranton, Victoria Obergh, Madison Goforth, Kavitha Ravi, Poornima Jayakrishna, Anisa Khan, Stephanie A. Boone, Charles P. Gerba, M. Khalid Ijaz, Frank Y. Xu, Karl Krupp, Purnima Madhivanan, Kerry K. Cooper

**Affiliations:** 1 School of Animal and Comparative Biomedical Sciences, The University of Arizona, Tucson, AZ, USA; 2 Public Health Research Institute of India, Mysuru, India; 3 Department of Environmental Science, The University of Arizona, Tucson, AZ, USA; 4 Global Research and Development for Lysol and Dettol, Reckitt Benckiser, Slough, England, UK; 5 Department of Public Health Practice, Policy and Translational Research, The University of Arizona, Phoenix, AZ, USA; 6 Department of Health Promotion Sciences, The University of Arizona, Tucson, AZ, USA; 7 BIO5 Institute, The University of Arizona, Tucson, AZ, USA

**Keywords:** built microbiome, household microbiome, microbiome, shotgun metagenomics

## Abstract

Characterizing the household bacterial microbiome allows for a stronger understanding of the various microbes to which a person is exposed every day in their home. Exploring household microbiomes in different countries around the world increases our understanding of the impact human behaviour might have on niche microbial communities in the house. The goal of this study was to use shotgun metagenomics to characterize the microbiome from ten locations around the house in ten different houses from three countries (Mysuru, India; Dubai, United Arab Emirates (UAE); and Tucson, USA). There was a significant difference in alpha diversity between the three countries (ANOVA, *P*<0.001), with homes in Mysuru, India, showing significantly higher bacterial diversity compared with those in Tucson, AZ, USA and Dubai, UAE. Beta diversity analysis of the homes found that bacterial communities differed significantly by geographic location [permutational multivariate analysis of variance (PERMANOVA), *P*=0.001] and, within cities, by household location (PERMANOVA, *P*=0.001), with geographic location explaining 5.3% and household location explaining 7.3% of variation. Household location was a strong driver of diversity within each of the three cities individually, accounting for 14–18% of microbiome variation. The highest levels of bacterial diversity across the three cities were found underneath the toilet rim and within bathroom and kitchen sinks. A core microbiome of classes *Actinomycetota* and *Gammaproteobacteria* was found in all homes in all three cities. Within each city, a core microbiome was identified at the species level within specific household locations. Many bacterial taxa found in the homes were a part of the human-associated phyla *Actinomycetota* (e.g. genera *Brevibacterium*, *Mycolicibacterium* and *Rhodococcus*), *Pseudomonadota* (e.g. genera *Acinetobacter, Moraxella, Paracoccus* and *Pseudomonas*) and *Bacillota* (genus *Streptococcus*), which is comparable to previous studies. The household microbiome is variable across different locations within the house and on a global scale. Factors such as human activity, cultural practices, climate and surface type and use may drive this diversity. Characterizing the household microbiome on a global scale allows for a better understanding of what drives microbial diversity, increasing our understanding of how microbial communities are shaped by the environment and how humans influence their dynamics, as well as any risks to human health that the built microbiome may potentially pose.

**Impact Statement**

This research contributes to the understanding of the built microbiome, specifically how it varies within houses, cities and across the globe. This can aid in our understanding of microbial dynamics in environments with heavy human influence and help develop and improve hygiene habits and products that are mindful of the existing microbiome.

## Data Summary

DNA sequence data from this research is publicly available on the NCBI’s Sequence Read Archive under BioProject PRJNA1416920. Data were analysed using Python and R code. Analysis protocols and information on software versions, packages and more can be found within the text and in the following GitHub repository: https://github.com/carolinescranton01/Global_Household_Microbiome. The authors confirm all supporting data, code and protocols have been provided within the article or through supplementary data files.

## Introduction

The indoor microbiome is a complex and multifaceted environment influenced by a myriad of both biotic and abiotic factors that can have major impacts on human health [1–3]. The microbial communities comprising the indoor microbiome are a joint result of direct and indirect factors such as the daily activities of the buildings’ occupants and the surrounding external environment [3, 4]. Estimates show society has spent increasing quantities of time indoors (65–90% of human activity occurring in enclosed buildings per day), especially in locations with higher degrees of socioeconomic development and under the circumstances of the SARS-CoV-2 global pandemic [3–6]. Humans are key contributors to the indoor microbiome, where micro-organisms can maintain viability for extended durations in aerosols or on fomites, where active proliferation or persistence may occur after introduction [3, 4, 7, 8]. Microbes inside may be transient or established, and survival varies between the surfaces and locations within the household and is considerably influenced by the duration and frequency of sanitation and cleaning efforts of the occupants [4, 8, 9]. As a result, the transmission and preservation of microbes within the built environment is complex and determined by a multitude of factors [3, 4].

The introduction and persistence of indoor microbiota rely on physiological determinants, including moisture, relative humidity, temperature, ultraviolet radiation, particle matter size, indoor/outdoor ratio of pollutants and particulates and heat and ventilation within the indoor space [3, 4, 8, 10]. Studies have shown that buildings in more industrialized locations like major cities tend to have reduced soil-derived microbes in the indoor microbiome compared to those in rural areas [2, 8, 11]. Thus, the composition of the indoor microbiome is not universally consistent. Globally, the built environment varies in size, building materials, ventilation, pollutant levels, proximity to green spaces and climate [4, 6, 8]. Equivalently, occupant behaviour disparities due to socioeconomic factors and cultural dissimilarities influence the composition of the indoor microflora [4, 6, 9]. Therefore, understanding the indoor household microbiome, how it varies in different areas of the household and spatially between households located within the same region and how it differs geographically between countries aids our understanding of how the built microbiome develops and what risks it can potentially pose to household occupants.

In residential environments, previous indoor microbiome analyses of touch surfaces and interior dust deposition have demonstrated that there may be a relatively common core of microbial phyla consisting of *Pseudomonadota*, *Bacillota* and *Actinomycetota* [1, 3, 7, 10, 12, 13]. These phyla are heavily associated with the natural human microbiome of the skin, gut, oral cavity and secretions [14, 15]. Studies in the USA [7, 14], India [13], the UK, Greece [9] and China [11], as well as a global review [1], examining the surfaces of kitchens, bathrooms and common living areas (living room or bedroom), frequently identified these ‘core’ phyla alongside members of the *Bacteroidota*. The variability and diversity of microbial taxa within these phyla, however, were dependent upon the site or location of sampling, where sites with moist environments typically harboured taxa different from those located on dry contact surfaces [3, 13]. Comparatively, locations frequently exposed to human-associated sources harboured different taxa from those infrequently touched or those in proximity to outdoor air deposition [1, 7, 10, 12]. Additionally, areas that lacked regular cleaning patterns typically showed greater microbial diversity [4]. A crucial reservoir for household microbes is surface dust, which can contain 500 to 1,000 unique species, according to some reports [9]. Analyses of the microflora of indoor dust have demonstrated that its composition is complex, containing microbes and microbial secretions, particulates of human and animal origin (such as skin cells or pet dander), outdoor particulates (from soil and plants) and indoor contributions (cooking, smoking and inorganics from cleaning products) [4, 8, 9, 12, 16]. Thompson *et al.* [9] demonstrated differences in taxa based on geographic location, where they observed a distinct country-specific microbiome variance between dust samples from the UK and Greece [9]. A similar trend was observed by Benton *et al.* [2], who found floor dust in Nogales (Sonora, Mexico) was more enriched with three different soil microbial genera when compared to dust from Tucson (Arizona, USA) despite a spatial separation of only 100 km and similar climates [2]. However, differences in the study could also be attributed to socioeconomic factors and the structure of the built environment [2].

It is important to understand the differences in microbial diversity across a range of household surfaces and geographic locations, and how factors such as socioeconomic and cultural differences impact the microbiome. This may present possible health implications for household occupants and the presence of potential pathogens, as microbe persistence and presence within indoor spaces has been associated with both protective and adverse health effects [4, 7, 9, 11]. There are few studies assessing the household microbiome in our three study locations – some were conducted in the USA, one was recently conducted in India and none in the United Arab Emirates (UAE) [10, 13, 17]. This study assessed the household bacterial microbiome in three major cities (population>500,000) around the globe – Mysuru (Karnataka, India), Dubai (UAE) and Tucson (Arizona, USA). Ten different sampling locations were assessed in ten houses per city, focusing on the kitchen and bathroom. The microbiome was analysed to determine dominant taxa across the locations, factors driving microbiome diversity and a potential core microbiome, both within the cities and across the same household location in different cities across the world.

## Methods

### Household sampling

Samples were collected from three geographic locations – Tucson (Arizona, USA – population est. 555,000 [18]), Mysuru (Karnataka, India – population est. 3,000,000 [19]) and Dubai (UAE – population est. 3,800,000 [20]). Participants (households) were recruited by local teams associated with the study in January (Mysuru), February (Tucson) and September (Dubai). These sampling months were chosen based on researcher availability for travel and local lab availability to conduct the necessary experiments. Households had between two and six adults and zero to three children as residents, with one to six bathrooms and two to six bedrooms. Study households were chosen based on connections with local collaborators who fit the above criteria. Households with pets were not excluded, but this was not considered in the presented analysis. Household occupants were present during sampling but stood away from sampling surfaces. They were not given advance notice (more than a day) of sampling and were instructed not to partake in extra cleaning in preparation for our sampling. Up to ten samples were collected from each household depending on availability, household construction and participant willingness to provide access (Table S1, available in the online Supplementary Material), for a total of 100 samples in Tucson and Mysuru and 89 samples in Dubai. Sampling locations included the kitchen counter surface, kitchen sink (surface and around but not inside the drain), coffee table surface, TV remote (all sides and buttons), showerhead, bathroom sink (surface and around but not inside the drain) and four locations on the toilet (all of which were standard western toilets); the seat (outside and inside), rim of the toilet (top and outside), toilet bowl above the water and under the toilet rim (inside the bowl). All households were in urban environments and had standard urban features such as closable windows, finished floors and no gaps within the household structure that could let environmental debris into the house. Samples were collected with dual-end swabs [Becton Dickinson (Franklin Lakes, NJ, USA), BBL CultureSwab] that were dipped in a sterile detergent solution (0.15 M NaCl with 0.1% Tween 20) [21] and then dragged across the sample surfaces for at least 15 s but no more than 30 s, rotating the swab during the sampling process. A specific surface area to swab was not specified for each household location, as there was too much variation between households and cities to standardize this aspect of collection. However, the samples were taken from the entire surface of a particular targeted household sample location (i.e. corners, edges, centre, etc.) to effectively sample all areas of the surface. Swabs were stored in their original sterile tubes and kept on ice in a cooler for less than 8 h prior to storing at −20 ˚C long term. Samples collected in Mysuru (*n*=100) were processed and sequenced on site at the Public Health Research Institute of India (PHRII) for the study. Samples from Tucson (*n*=100) were processed and sequenced in the Cooper lab (University of Arizona, Tucson, AZ). Samples collected in Dubai, UAE (*n*=89, as some households lacked the sampling locations of interest) were shipped on dry ice to the Cooper lab for processing and sequencing. In addition to household samples, information on the number of occupants living in each house (children and adults) and the number of times the household was cleaned per week was collected and considered in the presented analysis.

Prior to conducting the study, the study design for Tucson and Dubai was reviewed by the University of Arizona’s Institutional Review Board (IRB), which determined that, because no human-identifying data were collected during the study, it was exempt from IRB review. The study design for households in India was approved by the PHRII’s IRB Committee prior to sampling.

### DNA extraction

DNA extraction from each sample included removing one of the two swabs and extracting DNA using the DNeasy Powersoil Pro Kit (Qiagen) as per the manufacturer’s instructions, with one modification – samples were heated at 65 ˚C for 10 min after adding the first lysis buffer (CD1) [22]. DNA concentrations were measured using a Qubit fluorometer and the dsDNA High-Sensitivity Assay Kit (Invitrogen, Carlsbad, CA, USA). If the DNA concentration was deemed too low by the Qubit assay (less than 0.0005 μg ml^−1^), the second swab was used for DNA extraction to increase the yield.

### Oxford nanopore sequencing

Sequencing libraries in the Cooper laboratory were prepared using the Oxford Nanopore Rapid PCR Barcoding Kit 24 V14 [Oxford Nanopore Technology (ONT), SQK-RPB114.24, protocol version RBK_9176_v114_revK_27Nov2022] as per the manufacturer’s instructions. Sequencing libraries prepared at PHRII used the Oxford Nanopore Rapid Barcoding Kit 96 V14 (ONT, SQK-RBK114.96, protocol version RBK_9176_v114_revK_27Nov2022) according to the manufacturer’s protocols. This PCR-free library preparation kit was used as there were no thermocyclers available on-site at PHRII, so samples were sequenced multiple times to increase sequencing yield. Some samples from Tucson were prepared and sequenced using both the SQK-RPB114.24 and SQK-RBK114.96 library preparation kits. All samples were sequenced using ONT on MinION Mk1B devices (ONT) with R10.4.1 flow cells (ONT). Libraries were sequenced multiple times at both locations to increase the data yield from each sample and concatenated into a single sample per city, household and location. The concatenation of multiple reads across several runs likely minimized any batch effects; thus, traditional batch correction was not performed. However, sequencing kit chemistry was considered a potential confounding variable. Due to logistic issues in Mysuru and Dubai, we were unable to run negative controls with the specific library preparation kits used for the samples. To remediate this, negative control samples were run in Tucson using the same sequencing kits (SQK-RBK114.96 and RPB114.24), with triplicate samples of nuclease-free water (which was DNA extracted in an identical manner to other samples). This was done to assess the ‘kitome’, or any micro-organisms present in the kit reagents, for each sequencing method.

### Taxonomic analysis

Reads were generated using multiple sequencing runs to increase data yield and concatenated across runs to form FASTQ files for each sample (ten samples per house, ten houses per city, for all three cities; however, a few samples could not be collected in Dubai; final dataset *n*=278). Concatenated reads were filtered to remove low-quality sequences using Filtlong [23] (v0.3.1), with parameters set to filter reads to a minimum length of 1,000 bases and to discard the lowest 10% of reads based on quality. Table S2 shows read statistics for each sample before and after filtering. Reads were then analysed for taxonomic composition using Kraken2 [24] software (v2.3.1), using a database that contained all reference genomes from the NCBI database as well as all non-redundant sequences (nr database, as of 17 February 2023), which was created by the Cooper laboratory following the kraken2-build protocol for Kraken2 [24]. Kraken2 was run on all concatenated FASTQ files. A BIOM-format file was generated from Kraken2 reports using the kraken_biom software (v1.2.0) [25] and analysed as a phyloseq object in R with the phyloseq package (v1.52.0) [26]. After importing the data into R (v4.6.0) using RStudio (v2026.4.0.526), the data were filtered to include only bacterial taxa (which accounted for ~80.81% of the reads). This was done by removing all taxonomic assignments outside of the domain Bacteria. The remaining 19.19% of reads primarily belonged to the phyla Chordata and Arthropoda, which were excluded from the analysis. Samples were not rarefied to preserve rare taxa, but a decontamination analysis was conducted using the R package decontam [27], which identifies contaminants based on the prevalence and frequency of taxa in the negative control samples. Taxa identified as contaminants were excluded from further analysis. This decontaminated phyloseq object was used for the taxonomic, alpha diversity, beta diversity and core microbiome analyses, as described below using the following R packages: phyloseq, microbiome (v1.30.0) [28], microViz (v0.12.7) [29] and vegan (v2.7.3) [30].

### Bacterial diversity analysis

To assess alpha diversity across geographical and household sampling locations, the Shannon diversity index was used to capture both the richness and evenness of communities. The Bray–Curtis dissimilarity index was used to determine the beta diversity of the different samples in the study. The roles of the household locations, individual households, geographic location, sequencing kit, the number of household occupants and the number of times cleaned per week were assessed as potential drivers of beta diversity. Relative abundance of the top ten most abundant taxa in the dataset was calculated at the phylum and genus levels using the aggregate_taxa command from the R package microbiome. The core microbiome was analysed using the aggregate_taxa and aggregate_rare functions in the microbiome package for each city individually, across each household location within a particular city and across all three cities overall, at a prevalence level of 90% (taxa must be present in 90% of samples) and a detection level of 1% (taxa must be detected at a relative abundance of 1% or more in the sample to be included) for higher taxonomic orders (phyla, class, order and family) and 0.1% for the species level. Two species detected in the core microbiome analysis were known to share a large amount of their genomes – *Salmonella enterica* and *Shigella flexneri*. Reads mapped to these samples using Kraken2 were identified through NCBI taxIDs in the Kraken2 reports and were mapped to reference genomes for each species to ensure that these species were identified correctly. Analysis of microbiomes with bias correction (ANCOM-BC2) using the R package ANCOMBC (v2.14.0) [31] was conducted to identify phyla and genera with differential abundance across the household and geographic locations.

### Statistical analysis

Statistical analysis was conducted in R studio, with the significance threshold set at *P*<0.05. ANOVA tests were used to determine differences in alpha (Shannon) diversity between samples from different household locations in the same city and from the three cities using the microbiome package in R. Tukey’s post hoc tests were conducted using the stats [32] (v4.6.0) and multcompView [33] (v0.1.11) in R. Letters were used to represent significant differences – if two boxes on a box-and-whisker plot share a letter (i.e. a and ab), they do not differ significantly. Different letters, such as a and b, indicate a significant difference. Beta diversity was assessed using the Bray–Curtis dissimilarity index, and permutational multivariate analysis of variance (PERMANOVA) tests (conducted using the adonis2 function from the R package vegan) were used to determine which factors significantly drove microbiome beta diversity. To assess the role of geographic location on the beta diversity of the microbiome via PERMANOVA analysis, the following formula was used: (*dist_matrix~Geographic_Location*). To assess the role of household location, (*dist_matrix~Household_Location*) was used. To assess the interaction between the two factors, (*dist_matrix~Geographic_Location+Household_Location*) was used with the ‘*by=margin*’ variable (to test for the household effect after accounting for differences in geographic location, and vice versa). For the household number (to test if individual households have a unique microbiome that is driving diversity in the dataset), the number of occupants, sequencing kit and number of times cleaned per week, the formula remained the same (*dist_matrix ~ [variable of interest]*). The effect of household location was analysed further; the same formula was used, but the analysis was stratified by household number (*strata=metadata$house_number*) to determine whether diversity truly differs between household locations, accounting for the fact that some samples came from the same house. ANCOM-BC2 analysis used two formulas to compare taxa abundance across locations. Formulas for global analysis used the parameters *lib_cut=1000* to remove samples with less than 1,000 reads and *prv_cut=0.1* to exclude taxa present in less than 10% of the samples. The first analysis examined differential abundance across geographic locations while accounting for household location variance and included the variables *fix_formula=‘Geographic_Location+Household_Location’* and *group=‘Geographic_Location’*. The second examined differential abundance across household locations while accounting for geographic differences and included the variables *fix_formula=‘Geographic_Location+Household_Location’* and *group=‘Household_Location’*. City-specific ANCOM-BC2 tests were run on subsets of the full dataset containing only samples from each individual city, using the formula *fix_formula=‘Household_Location’* and *group=‘Household_Location’*. City-specific datasets were filtered prior to ANCOM-BC2 analysis to include only taxa present in 5% or more of the samples and to remove taxa with zero variance across the dataset. These ANCOM-BC2 formulas also used the parameters *lib_cut=0* and *prv_cut=0.05*; this was all done to account for sparsity in the data.

### Data visualization and availability

RStudio was used to subset and reorganize the data. Results were represented using visual plots generated using ggplot2 (v4.0.2) [34] in R using RStudio. The packages RColorBrewer (v1.1.3) [35], ggpubr (v0.6.3) [36] and ggplot2 were used to visualize the relative abundance of taxa, plot alpha diversity metrics as box-and-whisker plots and plot a principal components analysis of the taxonomic makeup of the samples using Bray–Curtis distances. The packages tidyr (v1.3.2) [37], data.table (v1.18.2.1) [38], dplyr (v1.2.0) [39], openxlsx (v4.2.8.1) [40] and writexl (v1.5.4) [41] were used to restructure data (add or change metadata, filter subsets of data and transpose data) throughout the analysis. Code used for data analysis can be found on GitHub (https://github.com/carolinescranton01/Global_Household_Microbiome). All raw sequence data have been submitted to NCBI’s Sequence Read Archive and are available under the BioProject accession PRJNA1416920.

## Results

### Data overview

The goal of this study was to examine the diversity of the household bacterial microbiome between different countries around the world. Households from three cities with at least 500,000 residents were sampled – Tucson, AZ, USA (~575,000 people); Dubai, UAE (~3.65 million people); and Mysuru, India (~3 million people). Before filtering, the mean number of sequences was 54991.9, with an average of 175,057,604 bases per sample. After filtering, these numbers dropped to 40,764.9 and 156,690,350, respectively. A total of 74.12% of reads were retained after filtering. The average number of bases above a Q30 Phred score increased slightly, from 11.07 to 11.68%. Decontamination analysis determined two taxa were contaminants – *Pantoea agglomerans* (NCBI taxID 549) and *Pantoea* spp. (NCBI taxID 53334). These taxa accounted for 9,840 reads (0.128% of the total dataset) and were removed from further analysis. The effects of sequencing depth and kit chemistry were evaluated as potential confounders in the dataset. Sequencing depth significantly explained geographic diversity but only accounted for roughly 3% of variation in the data (PERMANOVA, *P*=0.001, *R*^2^=0.0272). Kit chemistry was also a significant driver of microbiome diversity (PERMANOVA, *P*=0.001, *R*^2^=0.0519). However, kit chemistry was highly correlated with geographic location (Pearson’s chi-squared test, *χ*^2^=445.05, *P*<0.001); therefore, this effect was not considered in further analysis.

### Global

Alpha diversity significantly differed between the three cities, with Mysuru displaying the highest diversity, followed by Tucson and then Dubai (ANOVA, *P*<0.001). Within each city, alpha diversity differed significantly across household locations (ANOVA, *P*<0.01), but samples from under the toilet rim consistently displayed the highest Shannon diversity, regardless of geographic location (Fig. 1a). Beta diversity based on the Bray–Curtis dissimilarity index across all samples found that Mysuru samples clustered tightly, whereas Dubai and Tucson samples were more spread out on a principal coordinates analysis (PCoA) plot (Fig. 1b). PERMANOVA analysis revealed significant differences by geographic location (*P*=0.001, *R*^2^=0.05288), household location individually (*P*=0.001, *R*^2^=0.07294) and when household and geographic location were considered together (household: *P*=0.001, *R*^2^=0.07235; geographic: *P*=0.001, *R*^2^=0.05289). Additionally, beta diversity was significantly explained by the number of household occupants (*P*=0.001, *R*^2^=0.008) and the number of times the house was cleaned per week (*P*=0.001, *R*^2^=0.02949), but not by the individual houses (house number) themselves.

**Fig. 1.**
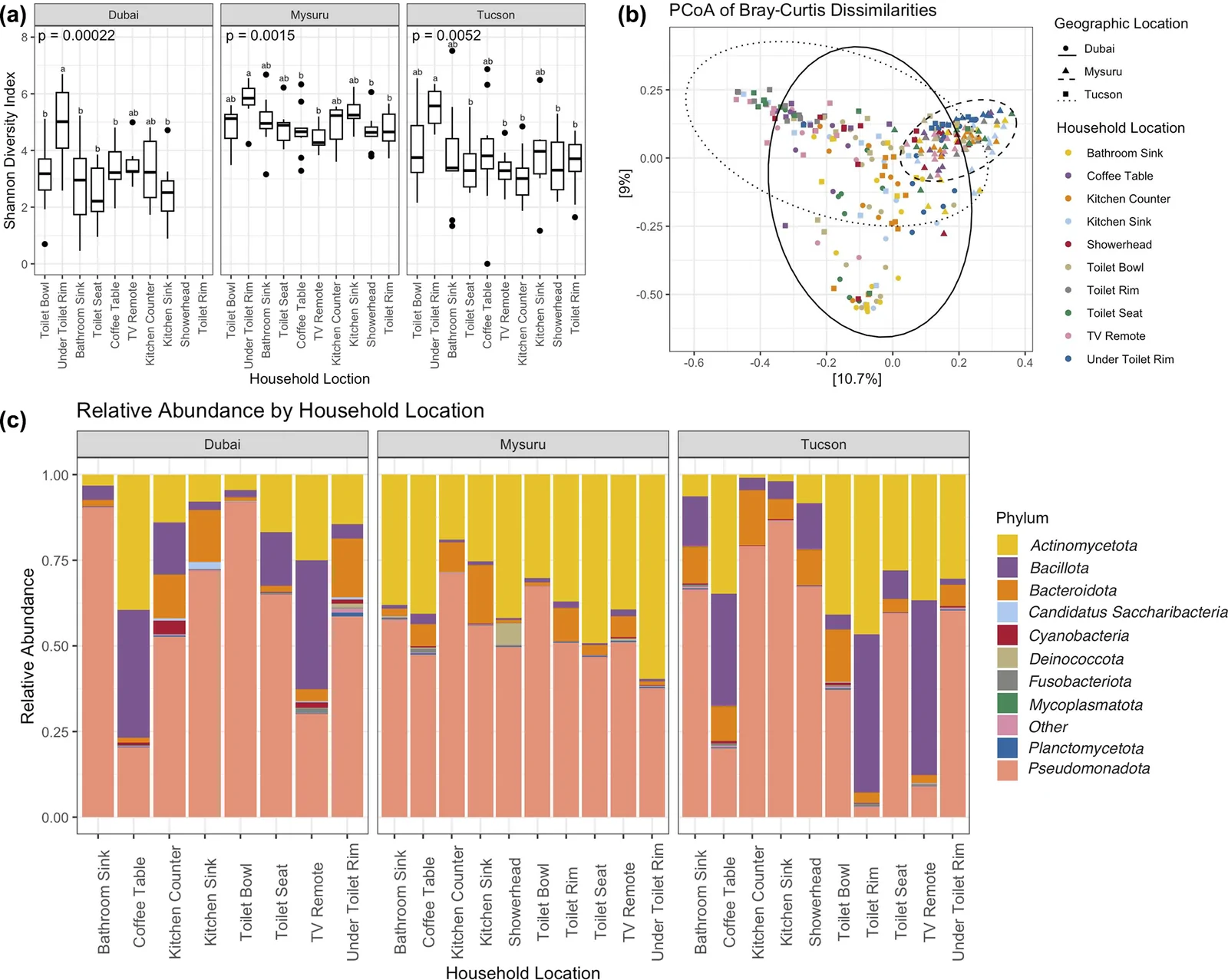
Taxonomic composition of the household microbiome across the three study locations. (a) Shannon diversity indices (alpha diversity) for the ten household locations in the three cities, with ANOVA calculations to determine differences in diversity between household locations in each city. (b) PCoA of Bray–Curtis distances (beta diversity). Samples are coloured by household location, and point shapes correspond to geographic locations. Ellipses are drawn around clusters by geographic location. (c) The relative abundance of the top ten most abundant phyla per household location within each geographic location.

The phylum *Pseudomonadota* had the highest relative abundance in the three cities overall, particularly in the kitchen sink samples of Dubai and Tucson, the bathroom sink samples from Dubai and the kitchen counter samples from Tucson. *Actinomycetota* was also found at a high relative abundance, particularly throughout Mysuru households and within Tucson toilet and TV remote samples. *Bacillota* was found at higher relative abundances on Dubai coffee tables and TV remotes and on Tucson coffee tables, toilet rims and TV remotes. *Bacteroidota* was also consistently found across the household microbiomes of all three cities (Fig. 1c). The household core microbiome for all three cities had no shared phyla, but at the class level, *Actinomycetes* and *Gammaproteobacteria* were present at a relative abundance of 1% or higher in 99% of samples (Table 1). Across the household locations and at the species level, a core microbiome of *Moraxella osloensis* existed on coffee tables, kitchen counters and kitchen sinks, where this taxon was found in 99% of samples at any abundance. The core species microbiome under the toilet rim included *Hyphomicrobiales* spp. and *Pseudomonas* spp., with the same detection parameters as specified above. All other household locations did not have a core microbiome across the three cities (Table 2).

**Table 1.** Core microbiota in each location

Taxonomic level	Dubai	Mysuru	Tucson	All location
Phylum*	*Pseudomonadota*	*Actinomycetota* *Pseudomonadota*	*Actinomycetota* *Bacillota* *Pseudomonadota*	None
Class^†^	*Gammaproteobacteria*	*Actinomycetes* *Alphaproteobacteria* *Betaproteobacteria* *Gammaproteobacteria*	*Actinomycetes* *Gammaproteobacteria* *Bacilli*	*Actinomycetes* *Gammaproteobacteria*
Order^‡^	None	*Burkholderiales* *Hyphomicrobiales* *Micrococcales* *Mycobacteriales Rhodobacterales* *Xanthomonadales* *Sphingomonales*	None	None
Family^§^	None	*Microbacteriaceae* *Paracoccaceae*	None	None

* Phylum level, prevalence=90%, detection=1%.

† Class level, prevalence=90%, detection=1%.

‡ Order level, prevalence=90%, detection=1%.

§ Family level, prevalence=90%, detection=1%.

**Table 2.** Core microbiome at the bacterial species level for each household sampling site in each location

Location	Dubai	Mysuru	Tucson	All location
Coffee table	*Corynebacterium tuberculostearicum, Staphylococcus epidermidis, Staphylococcus hominis*	*M. osloensis*	None	*M. osloensis*
TV remote	*Corynebacterium kefirresidentii, Cutibacterium acnes, Staphylococcus hominis*	*Brevibacterium casei*	*C. acnes, Staphylococcus epidermidis*	None
Kitchen counter	*M. osloensis*	*Epilithonimonas vandammei*	None	*M. osloensis*
Kitchen sink	*Acinetobacter junii*	*Paracoccus yeei, Salmonella enterica, Shigella flexneri,* *Streptomyces* spp.	*M. osloensis*	*M. osloensis*
Showerhead	*M. osloensis*	*Streptomyces* spp*.*	None	None
Bathroom sink	None	*Salmonella enterica*	None	None
Toilet seat	None	None	*M. osloensis*	None
Toilet rim	None	*M. osloensis*	*Staphylococcus epidermidis*	None
Toilet bowl	None	*E. vandammei, M. osloensis*	None	None
Under the toilet rim	*Pseudomonas aeruginosa, Hyphomicrobiales* spp.	*Mycobacteriaceae* spp., *Hyphomicrobiales* spp., *Mycolicibacterium* spp.	None	*P. aeruginosa, Hyphomicrobiales* spp.

Core microbiome at the species level was calculated using the following parameters: prevalence=90%, detection=0.1%.

The bacterial classes *Gammaproteobacteria* and *Actinomycetes* dominated most samples across all three cities. Other classes with higher relative abundances include *Alphaproteobacteria* (particularly in Mysuru) and *Bacilli* (abundant on Tucson and Dubai TV remotes and coffee tables) (Fig. S1a). Despite this, there was no core microbiome across the cities at the class level (Table 1). Dominant bacterial orders across the dataset include *Moraxellales*, *Micrococcales*, *Mycobacteriales* and *Hyphomicrobiales*, although no global core microbiome existed at this taxonomic level (Fig. S1b, Table 1). At the family level, many household locations across all cities were dominated by ‘other’ families, or those not found in the top ten most abundant in the dataset. However, the families *Moraxellaceae*, *Paracoccaceae*, *Weeksellaceae* and *Streptococcaceae* were found consistently across the dataset in almost all household locations in each city. Again, no core microbiome existed across the three cities at this level (Fig. S1c, Table 1).

The abundance of 23 phyla was significantly different across geographic locations. The most differentially abundant phyla included *Bacillota* [higher abundance in Dubai than Mysuru (LFC=2.22), higher abundance in Tucson than Dubai (LFC=1.08) and higher abundance in Tucson than Mysuru (LFC=3.30)], *Mycoplasmatota* [higher abundance in Dubai than Mysuru (LFC=1.68), slightly higher abundance in Tucson than Dubai (LFC=0.11) and higher abundance in Tucson than Mysuru (LFC=1.8)] and *Fusobacteriota* [higher abundance in Dubai than Mysuru (LFC=2.22), higher abundance in Tucson than Dubai (LFC=0.61) and higher abundance in Tucson than Mysuru (LFC=2.29)]. Of the top ten most abundant phyla in the dataset, eight were differentially abundant across the three cities. *Actinomycetota* was greatest in Mysuru, whereas Candidatus *Saccharibacteria* and *Pseudomonadota* were greatest in Dubai. *Bacillota*, *Bacteroidota*, *Cyanobacteriota*, *Fusobacteriota* and *Mycoplasmatota* were most abundant in Tucson. Seven of these eight phyla (all but *Actinomycetota*) were least abundant in Mysuru. Twenty-four phyla were also differentially abundant across the household locations – this included all ten of the most abundant phyla. Two phyla were most abundant on kitchen counters (*Pseudomonadota* and *Actinomycetota*), four on kitchen sinks (*Mycoplasmatota*, *Cyanobacteriota*, *Bacillota* and *Bacteroidota*), one on the toilet seat (Candidatus *Saccharibacteria*), one on the toilet rim (*Fusobacteriota*), one on the showerhead (*Deinococcota*) and one underneath the toilet rim (*Planctomycetota*). All phyla except *Bacillota* and Candidatus *Saccharibacteria* were least abundant on the TV remote; these two were least abundant under the toilet rim and on the coffee table, respectively.

A total of 887 genera differed significantly in abundance across the three geographic locations. The most significant taxa included *Shigella* (found 2.5-log fold higher in Mysuru than Dubai, 0.45-log fold higher in Tucson than Dubai and 2.08-log fold higher in Mysuru than Tucson), *Streptococcus* [found significantly higher in Dubai than Mysuru (LFC=2.59), higher in Tucson than Dubai (LFC=1.15) and higher in Tucson than Mysuru (LFC=3.75)] and *Cutibacterium* (found 1.56-log fold higher in Dubai than Mysuru, 1.78-log fold higher in Tucson than Dubai and 3.5-log fold higher in Tucson than Mysuru). Of the top ten most abundant genera, eight displayed significant differences in abundance across the three cities. The levels of the genera *Acinetobacter*, *Epilithonimonas* and *Moraxella* were most enriched in Dubai, whereas *Mycolicibacterium* and *Paracoccus* were most enriched in Mysuru. *Rhodococcus, Sphingobium* and *Streptococcus* were enriched in Tucson. Across household locations, 497 taxa were differentially abundant, including all ten of the most abundant genera in the dataset. Three genera were most enriched on kitchen counters (*Acinetobacter, Moraxella* and *Pseudomonas*), three on kitchen sinks (*Paracoccus, Chryseobacterium* and *Epilithonimonas*), three under the toilet rim (*Mycolicibacterium, Sphingobium* and *Rhodococcus*) and one in bathroom sinks (*Streptococcus*). Seven of these taxa were least enriched on TV remotes, one on kitchen counters, one under the toilet rim and one on coffee tables. Full ANCOM-BC2 results for the complete dataset can be found in Table S3.

### Mysuru, India

The alpha diversity between household locations in Mysuru significantly differed (ANOVA, *P*=0.0015). Samples collected from underneath the toilet rim displayed the highest diversity, which significantly differed from the lowest-diversity samples collected from the coffee table, TV remote, showerhead and toilet rim. The other locations (toilet bowl, bathroom sink, toilet seat, kitchen counter and kitchen sink) had moderate diversity, which was not significantly different from the highest- or lowest-diversity samples (Fig. 1a; Mysuru). Samples from Mysuru showed strong clustering relative to Dubai and Tucson samples, suggesting a stronger signal associated with geographic location (Fig. 1b). PERMANOVA analysis of the Bray–Curtis dissimilarities in Mysuru revealed that diversity was significantly driven by household location (*P*=0.001, *R*^2^=0.18327). Additionally, the house number was significant (*P*=0.005, *R*^2^=0.11621), and when household location was stratified by house number, this explained a high degree of microbiome beta diversity (*P*=0.001, *R*^2^=0.18327). The number of occupants did not significantly drive diversity (*P*=0.222, *R*^2^=0.01153), but the number of times cleaned per week and number of reads per sample did (cleaned per week: *P*=0.029, *R*^2^=0.04087, reads: *P*=0.001, *R*^2^=0.11662). The core microbiome in Mysuru contained seven orders (*Burkholderiales*, *Hyphomicrobiales*, *Micrococcales*, *Mycobacteriales*, *Rhodobacteriales*, *Xanthomonadales* and *Sphingomonales*) and two families (*Microbacteriaceae* and *Paracoccaceae*), which were found in 99% or more samples at a 1% abundance or greater (Table 1). A core microbiota existed across all household locations except for the toilet seat. This was composed of one to four species, including *M. osloensis*, *Brevibacterium casei, Salmonella enterica* and more, but there was no core species microbiome in Mysuru households overall (Table 2). Most genera found in Mysuru households were not part of the top ten most abundant, as denoted by a large ‘other’ proportion in Fig. 2a. Coffee table, TV remote and toilet seat samples were composed of similar proportions of taxa, with low levels of *Acinetobacter, Mycolicibacterium, Paracoccus* and *Moraxella*. Coffee table samples showed the highest levels of *Sphingobium* across Mysuru household sites. The kitchen counter and sink also showed similar taxonomic profiles, with higher levels of *Epilithonimonas* and *Paracoccus* than other sites. The showerhead had a large proportion of *Moraxella* reads, while the bathroom sink had elevated *Pseudomonas* compared to other locations. The toilet bowl samples were heavily dominated by *Acinetobacter*, which accounted for more than half of the bacteria detected at this location. Toilet rim samples had higher levels of *Chryseobacterium* than other locations but had a similar microbiome to coffee tables, TV remotes and toilet seats. Samples collected from under the toilet rim had the highest levels of *Mycolicibacterium* across the ten locations (Fig. 2a). Aligning with the PERMANOVA analysis, clustering can be seen by household location in the PCoA plot, where samples from the same household locations (under the toilet rim, kitchen sink and toilet seat) form distinct groups. Some clustering can be seen between multiple household locations (coffee table, TV remote and toilet seat), which agrees with the similar compositions observed in the relative abundance analysis (Fig. 2b). A total of 192 taxa displayed statistically significant differential abundance across the household locations in Mysuru, including eight of the ten most abundant genera in the dataset. *Acinetobacter, Epilithonimonas, Moraxella, Paracoccus, Pseudomonas, Rhodococcus* and *Sphingobium* were significantly enriched in kitchen sinks. *Mycolicibacterium* was enriched more on TV remotes than at any other location. *Acinetobacter* was depleted on the TV remote, whereas *Epilithonimonas, Moraxella, Paracoccus, Pseudomonas, Rhodococcus* and *Sphingobium* were depleted under the toilet rim. *Mycolicibacterium* was least enriched on the toilet rim. Genera enriched in kitchen sink samples were often also enriched in kitchen counters and toilet bowls. Table S4 provides all pairwise comparisons for all taxa with differential abundance across household locations.

**Fig. 2.**
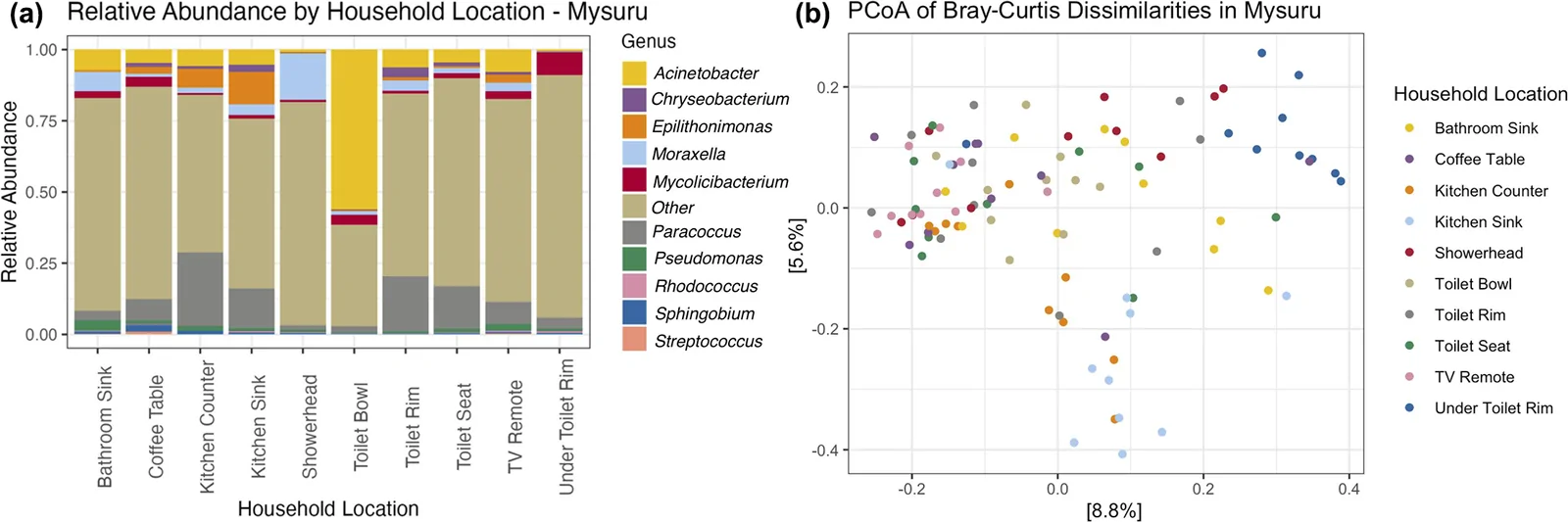
Taxonomic composition of samples from Mysuru, India. (a) Relative abundance of the top ten taxonomic genera from each household location. (b) PCoA of Bray–Curtis distances for Mysuru samples. Points are coloured by household location.

### Dubai, UAE

Significant differences in alpha diversity were observed across Dubai household locations (ANOVA, *P*=0.00022). The highest Shannon diversity was observed under the toilet rim, which was significantly higher than the toilet bowl and seat, the bathroom sink, the coffee table and the kitchen sink. Shannon diversity on the TV remote and kitchen counter did not significantly differ from the other locations (Fig. 1a; Dubai). Beta diversity in Dubai was significantly driven by household location (PERMANOVA, *P*=0.001, *R*^2^=0.14691), house number (PERMANOVA, *P*=0.037, *R*^2^=0.12914) and household location stratified by house number (PERMANOVA, *P*=0.001, *R*^2^=0.14691). The number of sequencing reads was also significantly correlated with diversity (PERMANOVA, *P*=0.001, *R*^2^=0.04673). The number of household occupants and the number of times the house was cleaned per week were not significant drivers of beta diversity (PERMANOVA, *P*=0.28 and *P*=0.376, respectively). Samples from Dubai did not cluster as strongly on a PCoA plot of Bray–Curtis dissimilarities compared to Mysuru but clustered roughly equally to samples from Tucson, suggesting a wider degree of diversity in the microbiomes from these locations (Fig. 1b). A core microbiome in Dubai only existed at the phylum and class taxonomic levels, containing the phylum *Pseudomonadota* and the class *Gammaproteobacteria* (Table 1). Across the household locations in Dubai, a core species microbiome was found on the coffee table (*Corynebacterium tuberculostearicum, Staphylococcus epidermidis* and *Staphylococcus hominis*), on the TV remote (*Corynebacterium kefirresidentii, Cutibacterium acnes* and *Staphylococcus hominis*), in the kitchen sink (*Acinetobacter junii*), under the toilet rim (*Pseudomonas aeruginosa* and *Hyphomicrobiales* spp.) and on the kitchen counter and showerhead (both contained *M. osloensis*) (Table 2). The Dubai household microbiome was dominated by *Moraxella*, which accounted for roughly 50% of all reads from the bathroom sink, toilet bowl and toilet seat and was also found in high levels under the toilet rim, in the kitchen sink and on the TV remote. *Acinetobacter* was dominant in the bathroom and kitchen sink, kitchen counter and toilet bowl. Other notable taxa in Dubai households included *Streptococcus* (abundant on the TV remote) and *Epilithonimonas* (found in the kitchen sink) (Fig. 3a). Some clustering by household location can be observed in Fig. 3b, particularly in samples from under the toilet rim and from the coffee table. Points are less clustered than those from Mysuru, suggesting a more diverse microbiome, which is reflected by variability in the genera across the household locations in Fig. 3a. In Dubai, 122 genera displayed differential abundances across the household locations. Of the top ten most abundant genera, nine were significantly enriched in kitchen sinks (*Acinetobacter, Chryseobacterium, Epilithonimonas, Moraxella, Mycolicibacterium***,** *Paracoccus, Pseudomonas, Sphingobium* and *Streptococcus*). Four of these taxa were least enriched on TV remotes (*Acinetobacter, Chryseobacterium, Epilithonimonas* and *Moraxella*), four were least enriched on toilet seats (*Paracoccus, Pseudomonas, Sphingobium* and *Streptococcus*), while one taxon was least enriched in toilet bowls (*Mycolicibacterium*). Highly enriched genera in kitchen sinks were also highly enriched on kitchen counters, TV remotes and coffee tables, suggesting a kitchen or living room profile of the household microbiome that differed from bathroom spaces. More information on differential abundances of bacterial genera in Dubai can be found in Table S4.

**Fig. 3.**
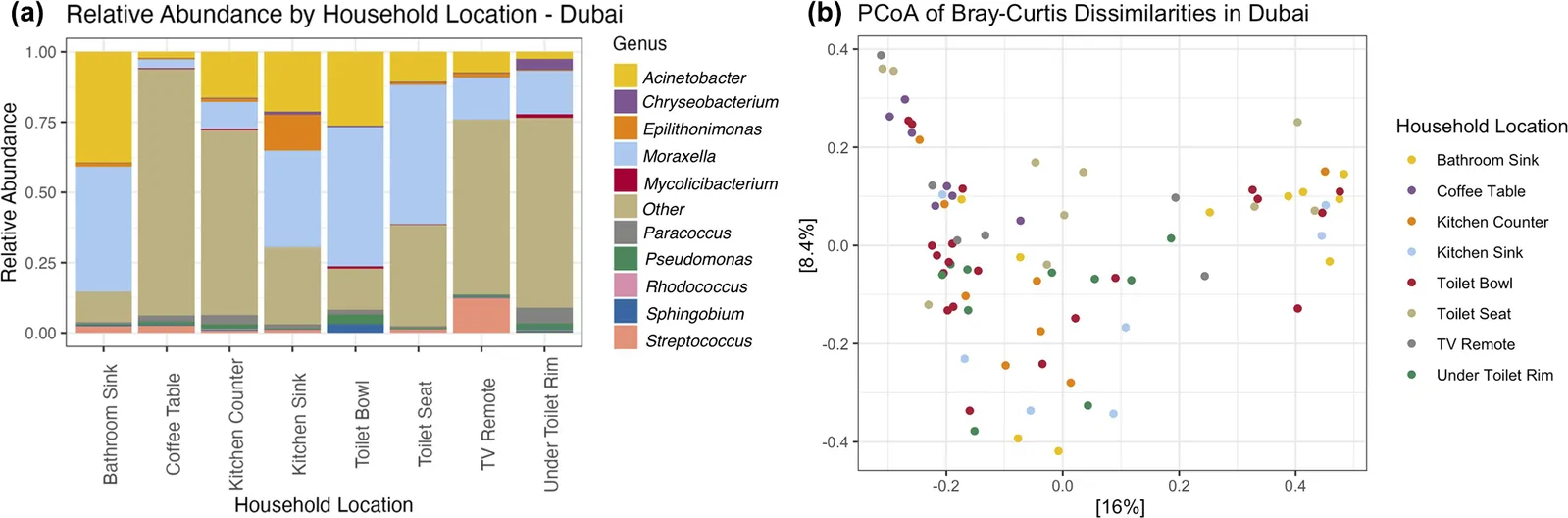
Taxonomic composition of samples from Dubai, UAE. (a) Relative abundance of the top ten taxonomic genera from each household location. (b) PCoA of Bray–Curtis distances for all samples collected in Dubai. Points are coloured by household location.

### Tucson, Arizona, USA

Alpha diversity significantly differed across household locations in Tucson (ANOVA, *P*=0.0052). Shannon diversity was significantly higher under the toilet rim compared to the toilet seat, TV remote, kitchen counter, showerhead and toilet rim. The toilet bowl, bathroom sink, coffee table and kitchen sink did not differ in Shannon diversity from that at the other household locations (Fig. 1a; Tucson). Statistical analysis of beta diversity in Tucson via PERMANOVA tests revealed that microbiome diversity is significantly driven by household location (*P*=0.001, *R*^2^=0.15426), house number (*P*=0.012, *R*^2^=0.10636) and household location stratified by house number (*P*=0.001, *R*^2^=0.15426). The number of occupants in the house was not a significant driver of diversity (*P*=0.084, *R*^2^=0.01265), whereas the number of times cleaned per week was significant (*P*=0.045, *R*^2^=0.03644). The number of reads per sample also significantly explained the beta diversity (*P*=0.001, *R*^2^=0.05011). Similar to Dubai samples, some clustering can be seen on a PCoA plot, with Tucson samples generally grouped together; however, this signal is weaker than that of Mysuru (Fig. 1b). A core microbiome in Tucson households was found only at the phylum (*Actinomycetota*, *Bacillota* and *Pseudomonadota*) and class (*Actinomycetes*, *Gammaproteobacteria* and *Bacilli*) taxonomic levels (Table 1). Across household locations in Tucson, a core microbiome of bacterial species was detected on the TV remote (*C. acnes* and *Staphylococcus epidermidis*), in the kitchen sink and on the toilet seat (both contained only *M. osloensis*), on the toilet rim (*Staphylococcus epidermidis*) and under the toilet rim (*Streptomyces* spp.) (Table 2). At the genus level, Tucson households displayed more variation among locations than those in Dubai and Mysuru. The coffee table and toilet rim were dominated by ‘other’ genera outside of the top ten, although both had detectable levels of *Streptococcus*. The TV remote was similar and had the largest proportion of *Streptococcus* across the ten locations. The bathroom sink, showerhead and toilet seat had elevated *Moraxella* levels, whereas the kitchen counter was dominated by *Acinetobacter. Rhodococcus* was detected at higher levels than in other locations (including in the other cities) on the toilet bowl and under the toilet rim samples from Tucson. *Pseudomonas* was also more common in Tucson, with low levels on the bathroom sink, coffee table, kitchen counter, kitchen sink and under the toilet rim. *Mycolicibacterium*, *Paracoccus* and *Sphingobium* were elevated in the toilet bowl and under the toilet rim samples (Fig. 4a). Some clustering can be seen in Tucson samples when plotted on a PCoA plot. The under the toilet rim samples cluster strongly, while TV remote and toilet rim samples are closely associated, along with some overlap from coffee table and kitchen sink samples (Fig. 4b). A total of 123 taxa had significant differential abundance across the household locations, which included all ten of the most abundant genera in the dataset. *Acinetobacter, Chryseobacterium* and *Streptococcus* were most enriched on kitchen counters, whereas *Sphingobium, Mycolicibacterium, Paracoccus* and *Rhodococcus* were most enriched under the toilet rim. *Moraxella* was enriched on coffee tables, *Epilithonimonas* in kitchen sinks, and *Chryseobacterium* on TV remotes. Bathroom sinks were the least enriched for all ten genera. Genera most enriched on kitchen counters were often highly enriched in kitchen sinks (and vice versa), suggesting a kitchen-specific niche. Complementary to this, genera most enriched under the toilet rim often showed enrichment (compared to other locations) at other bathroom locations, such as toilet bowls. More information on differentially abundant genera in Tucson can be found in Table S4.

**Fig. 4.**
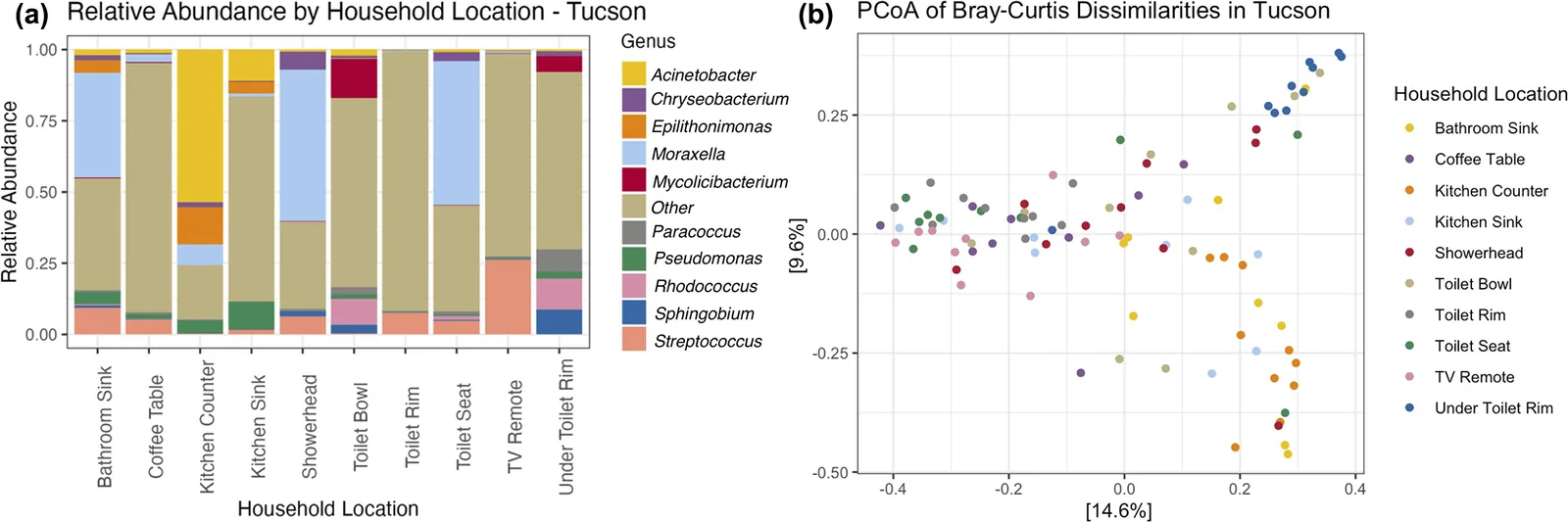
Taxonomic composition of samples from Tucson, Arizona, USA. (a) Relative abundance of the top ten taxonomic genera from each household location. (b) PCoA of Bray–Curtis distances for all samples collected in Tucson. Points are coloured by household location.

## Discussion

The household microbiome varied across locations within the house as well as between three global cities. Overall, it significantly varied among the three cities and across household locations. Within each city, the microbiome differed across different household locations, suggesting that household environmental roles like surface type and use may drive the microbiome composition. This is supported by the lack of a core microbiome across each city (particularly at lower taxonomic levels like species), and the presence of a core microbiome at some household locations, albeit a small one. Within each city, human-associated factors, including the number of occupants in the house, the number of times that the household was cleaned per week, and variation between each individual house, explained diversity in Mysuru and Dubai and, to a lesser extent, in Tucson. These findings suggest that the household microbiome is likely driven by a range of factors, including environment-associated variables like surface type and use and human-associated variables like cleaning habits.

The most abundant phyla identified in this study included *Pseudomonadota*, *Actinomycetota* and *Bacillota* (lesser so in Mysuru), aligning with previous global studies of built environments [16, 42, 43]. These taxa are highly associated with the human microbiome. *Actinomycetota* dominates the respiratory, skin and gut microbiome, whereas *Bacillota* is found abundantly in the mouth and respiratory tract, along with *Pseudomonadota* [13–15, 44, 45]. Many spore-forming and soil-associated bacteria are in the *Bacillota* and *Pseudomonadota* phyla and have been associated with dust in desert areas and within households [44, 46, 47]. Previous research has found these phyla to be common in indoor environments, along with *Bacteroidota* [1, 3, 7, 10, 12, 13], which was found at 1–25% relative abundance in each of the geographic locations in this study. Considering that the microbiome of built environments has been shown to shift from environmental microbes to human-related microbes as the length of inhabitance increases [48], the high prevalence of human-associated bacteria in the microbiome in this study and others is consistent with this information. Knowing that globally, the microbiome of the built environment is dominated by human-associated taxa, studying their roles and genes may aid our understanding of how the microbiome of the built environment impacts human health, as these microbes can live on and within humans. Our study found more variation in the built microbiome at lower taxonomic levels. *Actinomycetes* and *Gammaproteobacteria*, two human-associated taxa, were found to be core members across all samples from the study, which aligns with findings from the USA, India and China [1, 7, 10, 12].

The only published household microbiome study from India found *M. osloensis* as a core species in the microbiome – these findings are reflected in our study across some household locations in Mysuru [10]. Another study from the USA found similar bacterial families to those in our Tucson data being most abundant in the kitchen, such as *Moraxellaceae* (highly abundant on Tucson kitchen counters, showerheads and toilet seats, throughout Dubai and in toilet bowls in Mysuru) and *Streptococcaceae*, commonly associated with the human skin microbiome, on the TV remote (which was also found in our study, along with an increased abundance on Dubai TV remotes) [10, 13]. In contrast, another study that examined the household microbiome in five countries in Europe found different core and abundant taxa, including families like *Yersiniaceae* and genera such as *Bacillus* and *Enhydrobacter*, whereas these taxa were hardly detected, if at all, in our analysis [43]. Considerable differences in the microbiome were observed across geographic space and different household locations, suggesting that the microbiome composition is determined by a variety of factors related to the location, surface use and type and habits of household occupants.

Geographic location alone and in conjunction with household location significantly explained a small amount of variance in the household microbiome (5.289%). This trend may be stronger in Mysuru, as a tighter clustering of samples from this location can be observed from the PCoA plot in Fig. 1b. ANCOM-BC2 analysis revealed 23 differentially abundant phyla and 887 differentially abundant genera, including the 10 most abundant phyla and genera in the dataset, across the three global sites, suggesting a distinct geographic signal in the microbiome composition between cities. Visually, the taxonomic composition in each location is variable and inconsistent at all taxonomic levels, although Tucson and Dubai may exhibit more similar microbiomes to each other than to Mysuru. Tucson and Dubai are geographically separated by 8,370 miles across the globe but share similar climates, which are hot and dry [classified as arid, desert and hot (BWh) by the Köppen–Geiger climate system], whereas the climate in Mysuru is considered arid, steppe and hot (BSh) [49]. Climate was shown to strongly drive the indoor microbiome composition in a study by Wang *et al*. [50], so this is a possible explanation for the differences in bacterial diversity between the two climate types. Another potential driver of diversity between the household locations would be behaviour-associated factors, as human behaviour has been shown to shape the microbiome [48]. Factors such as when surfaces were last cleaned, used or touched by an individual living in the house, as external stimuli may momentarily impact the microbiome, even if it normally maintains a stable composition [42]. Although the number of occupants and times cleaned per week did not significantly explain microbiome diversity on a global scale, the number of times the house was cleaned per week was significant in Mysuru (0.4087% of variation) and Tucson (0.03644% of variation). Additionally, variation between houses (assessed with the ‘house number’ variable) significantly explained microbiome diversity in all three cities, accounting for 10.636% (Tucson), 11.621% (Mysuru) and 12.914% (Dubai) of variation in the microbiome. Additionally, it has been observed that lower socioeconomic status leads to a more diverse gut microbiome [51]. Given the strong impact that the human microbiome has on shaping the built microbiome, socioeconomic status could also play a subtle role in shaping the built microbiome. Other human behaviours, such as using air purifiers and Heating, Ventilation, and Air Conditioning (HVAC) systems, opening windows (many households in Mysuru had the windows open during sample collection, whereas in Tucson and Dubai they were closed), cultural practices and different types of cleaning products used throughout the house may also influence the household microbiome, although these variables were not collected in this study. Other household microbiome research has found that cultural practices, socioeconomic status, climate and hygiene habits play a large role in shaping the built microbiome [50]. This research provides evidence that geographic location plays a small role in household microbiome variation and that household-specific human activity may be influencing a greater degree of diversity in the microbiome. Cultural and household-specific habits should be explored further as potential drivers of the microbiome.

PERMANOVA analysis revealed that, on a global scale, the largest driver of microbiome diversity was household location, which alone explained 7.294% of variation in the microbiome. This was supported within each country, where household location explained 18.327% of microbiome variation in Mysuru, 14.691% in Dubai and 15.426% in Tucson (both when stratified by house number to account for variation between houses and alone). Specific genera were found to associate with household locations in all three cities, but these patterns were not consistent across the geographic locations. The sampled locations have diverse surface types, including plastic, ceramic, marble, stainless steel, wood and more, and are used for a wide range of human activities daily. Moisture levels could potentially drive microbiome composition, as these are heavily determined by surface type and use. Previous studies found that surface material and moisture levels influenced the diversity of microbes and even the presence and survivability of pathogens in a specific location [3, 13, 52, 53]. Our data reflect this through significantly raised alpha diversity under the toilet rim, an environment that remains moist and humid almost all the time, as well as elevated alpha diversity in the kitchen and bathroom sinks. The genera found under the toilet rim at higher rates than in other locations include *Chryseobacterium* and *Moraxella* (Dubai), *Rhodococcus* and *Sphingobium* (Tucson), *Mycolicibacterium* (Tucson and Mysuru) and *Paracoccus* (Dubai, Tucson and Mysuru). These genera are often soil- or environmental-associated taxa and can cause opportunistic infections, potentially posing a risk to household occupants [54–59]. On kitchen and bathroom sinks, *Epilithonimonas* (Mysuru and Tucson), *Acinetobacter* (Dubai, Mysuru and Tucson), *Moraxella* (Dubai and Mysuru), *Streptococcus* (Tucson) and *Pseudomonas* (Tucson) were more abundant. These taxa are also primarily found in the environment except for *Streptococcus*, which is part of the human flora [60–63]. It is likely that soil bacteria can colonize and persist in moist areas around the house due to the abundance of water and nutrients in these areas, while human skin-associated bacteria may struggle due to the high moisture content and lack of human-associated nutrients from sweat and skin oils [64, 65]. *Acinetobacter, Epilithonimonas* and *Pseudomonas* were enriched on kitchen surfaces compared to other locations in all three locations – these taxa are often associated with the environment [60] and could potentially be introduced into these household locations through food items (vegetables and meat). *Acinetobacter, Chryseobacterium* and *Moraxella* were found to be enriched in kitchen sponges (a moist and nutrient-rich environment) [52] – our analysis found these taxa to be enriched in kitchen spaces throughout the global household microbiome. Interestingly, taxa enriched in kitchen sinks were not highly enriched in bathroom sinks, suggesting that surface use (both locations are sinks) is not the only driver of microbiome composition. Lax *et al*. found that repeated wetting of a surface led to reduced microbial diversity [66] – our toilet bowl and showerhead data agree with this, as water routinely passes over these surfaces but does not pool, allowing the surface to dry and decreasing survivability for less ‘hardy’ microbes as they are rinsed off. These locations were colonized with similar bacteria as the other locations but had lower alpha diversity than most other locations, suggesting a less diverse microbiome. This idea is further supported through the differential abundance analysis, which found that the genera *Acinetobacter*, *Paracoccus, Rhodococcus* and *Sphingobium* were often enriched in both kitchen sinks, bathroom sinks and toilet bowls – three locations where water passes over the surface but does not pool. These genera may be better adapted to this niche. Differential abundance analysis did not identify any groups of organisms that were clearly enriched in the toilet environment overall – this may be due to the wide range of microenvironments on a toilet (e.g. under the rim is much more protected, moist and often is cleaned less, whereas the toilet seat and rim may be cleaned often and are very dry surfaces). At the phylum level, Mysuru had a larger proportion of *Deinococcota* on the showerhead than any other sample – the high degree of resilience traits in this phylum may help it persist in the showerhead environment [67]. This research suggests that the strongest driver of the household microbiome is surface type and use and that individual households may harbour a specific microbiome due to cultural, socioeconomic and hygiene habit differences between houses. These factors are stronger determinants of the household microbiome than the specific global location where a house is situated.

This study has several important limitations. Firstly, several household locations sampled in Tucson and Mysuru were not sampled in Dubai households due to limited access, meaning that exact comparisons could not be made across all ten household locations in the three cities. Addressing this inconsistency would provide a clearer understanding of the taxa found in different household locations across the globe. Despite this, most of the locations were collected consistently across the 30 houses, so this issue impacted only a few sites. Additionally, due to differences in the capacities of the lab in Tucson versus Mysuru, different sequencing kits were used, which may impact the depth and breadth of the sequenced data. To address this, most samples were sequenced multiple times to get a standard number of reads per sample across the dataset. However, this does not fully reduce the bias between different sequencing library preparations. Additionally, the sequencing kit strongly confounded geographic location; therefore, the effects of the sequencing kit on the microbiome could not be determined or distinguished from geographic location effects. Negative controls were not sequenced in Mysuru and Dubai due to logistical issues. Although they were processed using the kits from all three locations in Tucson, the true ‘kitome’ may be misrepresented by these negative control samples, as they were not sequenced in the Mysuru and Dubai labs. Analysis focused on relative abundance of taxa, which may misrepresent the true abundance of certain taxa within each sample. This study did not analyse cleaning, cultural habits or other factors, such as flooring type or presence of pets in the houses. Information such as how often the counters and bathroom were cleaned, whether windows were kept open or closed, if pets are tracking in dirt from outside and more could add valuable context to the microbiome data. This could potentially explain why certain taxa were present in some locations but not others and explain some of the influence of human activity on bacterial diversity. We only sampled houses with western-style toilets (ceramic sitting toilets, as opposed to squat toilets) and indoor kitchens, two features that may not be common globally and may harbour very different bacterial profiles. The samples were collected in different months of the year (winter in Tucson and Mysore and early fall in Dubai), so seasonal differences could impact the microbiome. Finally, two of the three study locations had very similar climates (Tucson and Dubai), so sampling another location with a different climate could yield data that encompassed the household microbiome more wholly across the globe. To improve this study, more information should be collected on household habits to identify any influential lifestyle variables on the microbiome. The sample collection should be done at the same time of year, and additional study locations with varying climates could be added to increase the scope of the analysis. Finally, further studies could look at the functional capabilities of the microbiome and could assess changes in the microbiome over time, in contrast to our cross-sectional sampling.

The indoor household microbiome is dynamic and diverse, and the taxa that exploit this niche are determined by surface use and type, human and geographic factors. The household microbiome is largely influenced by the presence of humans in the house, shown by the most abundant phyla in the built microbiome being human-associated, which is consistent across many global cities, including those in this study [1, 3, 7, 10, 12, 13, 15, 45]. The microbiomes of different locations throughout the house were more variable, yet similarities were seen between the microbes found across the house in Dubai and Tucson, which have similar climates. Similar taxa were also found in household locations that experience the same moisture conditions. A great number of external factors are likely driving the diversity in the household, such as climate, cleaning regimes and different cultural practices. Future studies focused on the influence of human habits or control over some aspects that could potentially influence the microbiome, such as the presence of air conditioning in the household, are necessary to decipher what factors are driving the composition of the microbiome in human-occupied environments. Overall, our study found that the household bacterial microbiome in Tucson, Mysuru and Dubai was composed of human-associated phyla, similar to what was found in other cities during previous studies. However, the locations within houses in each city had more distinct bacterial communities at the genus level, which may be selected by the niche of the specific location in the house. Future research should investigate how targeted, surface-specific hygiene interventions (bidirectional hygiene or ‘bygiene’ approaches) influence the fate, survivability and ecological balance of the microbiome to mitigate risks from opportunistic pathogens while preserving a healthy indoor microbiome [68]. Although similarities can be drawn between household locations in one city and another, more research must be conducted to determine the specific factors, such as cultural practices, surface types, climate and cleaning and hygiene habits, that drive the composition of the microbiome across the house. Characterizing the microbiome of the household allows for an assessment of the risks to public health, as humans are exposed to the household microbiome on a daily basis, as well as provides evidence on what cultural practices, surface types and human activities influence microbial communities and their persistence in the environment.

## Supplementary material

10.1099/mgen.0.001861 Supplementary Material 1.

10.1099/mgen.0.001861 Fig. S1.

10.1099/mgen.0.001861 Table S1.

10.1099/mgen.0.001861 Table S2.

10.1099/mgen.0.001861 Table S3.

## Abbreviations

ANCOM-BC: analysis of microbiomes with bias correction
HVAC: Heating, Ventilation, and Air Conditioning
IRB: Institutional Review Board
ONT: Oxford Nanopore Technology
PCoA: principal coordinates analysis
PERMANOVA: permutational multivariate analysis of variance
PHRII: Public Health Research Institute of India
UAE: United Arab Emirates
